# Metastatic Epithelioid Trophoblastic Tumor Following Incomplete Methotrexate Therapy for Ectopic Pregnancy: A Case Report and Multimodal Management

**DOI:** 10.7759/cureus.105635

**Published:** 2026-03-22

**Authors:** Rona Ting

**Affiliations:** 1 Obstetrics and Gynaecology, Logan Hospital, Meadowbrook, AUS

**Keywords:** chemotherapy, ectopic pregnancy, epithelioid trophoblastic tumor, gestational trophoblastic neoplasia, hysterectomy, multimodal management, pulmonary metastases, β-human chorionic gonadotropin

## Abstract

Gestational trophoblastic neoplasia (GTN) comprises a spectrum of rare placental trophoblastic malignancies, including choriocarcinoma, placental site trophoblastic tumor, and the particularly uncommon epithelioid trophoblastic tumor (ETT). Unlike other GTN subtypes, ETT arises from chorionic-type intermediate trophoblasts and is characterized by relative resistance to chemotherapy, often necessitating primary surgical management. We report the case of a 33-year-old G6P5E1 woman with prior methotrexate treatment for suspected ectopic pregnancy who presented one year later with persistent abnormal uterine bleeding and markedly elevated serum β-human chorionic gonadotropin (β-hCG) of 36,477 IU/L. Imaging revealed a large uterine mass with multiple pulmonary nodules. Her β-hCG tumor marker was 177 IU/L, raising suspicion for trophoblastic neoplasm despite inconclusive initial curettage findings. Based on the International Federation of Gynecology and Obstetrics (FIGO) 2000 staging system and World Health Organization (WHO) prognostic scoring system, she was classified as FIGO Stage III with a WHO score of 9 (high-risk GTN). Total laparoscopic hysterectomy with bilateral salpingectomy confirmed metastatic ETT. Multi-agent chemotherapy with EMA-EP (etoposide, methotrexate, actinomycin-D, etoposide, and cisplatin) was initiated, resulting in a marked decline in β-hCG. The patient remains under ongoing multidisciplinary monitoring. This case highlights the diagnostic challenges of ETT in the setting of atypical β-hCG levels, its metastatic potential, relative chemoresistance, and the importance of early histopathologic confirmation and individualized management guided by FIGO staging and WHO prognostic scoring to optimize outcomes.

## Introduction

Gestational trophoblastic neoplasia (GTN) is a rare group of malignancies arising from placental trophoblastic tissue, encompassing choriocarcinoma, placental site trophoblastic tumor (PSTT), and epithelioid trophoblastic tumor (ETT) [1,2]. ETT is a particularly uncommon subtype originating from chorionic-type intermediate trophoblasts and is recognized as a distinct clinicopathologic entity with unique histologic and clinical features [3-5].

Persistent elevation of serum β-human chorionic gonadotropin (β-hCG) is a sensitive biomarker used for diagnosis, monitoring, and surveillance in GTN. However, ETT typically produces lower β-hCG levels than choriocarcinoma [3-5]. Our patient presented with markedly elevated β-hCG, which is unusual for ETT and highlights the importance of clinical vigilance in atypical cases.

GTN can develop after any type of pregnancy event, including molar pregnancy, spontaneous abortion, term pregnancy, and, rarely, ectopic pregnancy [6-8]. Early recognition and risk stratification are critical to guide management and improve outcomes.

The International Federation of Gynecology and Obstetrics (FIGO) 2000 staging system and the modified World Health Organization (WHO) prognostic scoring system remain the cornerstone of risk stratification in GTN [1,7]. Patients with WHO scores ≥7 are classified as high-risk and require intensive multimodal therapy [1].

While most GTNs respond favorably to chemotherapy with high cure rates, ETT is relatively chemoresistant, often necessitating surgical management, particularly hysterectomy, in patients with localized disease or completed childbearing [4-10]. Metastatic ETT requires a combination of surgery and multi-agent chemotherapy to optimize outcomes [4,9,10].

Here, we report a rare case of metastatic ETT in a patient with prior incomplete methotrexate therapy for ectopic pregnancy, notable for an atypically high serum β-hCG level for ETT, emphasizing the diagnostic challenges, the significance of FIGO staging and WHO prognostic scoring, and the role of multidisciplinary management in achieving optimal outcomes [1-5].

## Case presentation

A 33-year-old G6P5E1 woman presented to the emergency department on August 18, 2025, with abnormal uterine bleeding and a positive serum β-hCG level. Her obstetric history included five vaginal births, with the last delivery four years prior. She had no significant past medical or surgical history.

In March 2024, she was diagnosed with a pregnancy of unknown location and treated presumptively for ectopic pregnancy with intramuscular methotrexate. Her treatment course included a first dose of methotrexate on March 15, 2024, with a β-hCG of 626 IU/L, a second dose on April 2, 2024, with a β-hCG of 570 IU/L, and a last documented β-hCG of 599 IU/L on April 12, 2024, before she was lost to follow-up.

Over the following year, she experienced persistent abnormal uterine bleeding and irregular cycles, for which no investigations had been initiated. She consulted her general practitioner four months before presentation to our center. A β-hCG level of 13,700 IU/L and pelvic ultrasound excluded intrauterine and extrauterine pregnancy at that time. Despite these findings, she did not attend further follow-up.

On presentation to our center, the patient was alert and hemodynamically stable, with minimal vaginal bleeding. Vital signs were within normal limits, and the cardiovascular and respiratory examinations were unremarkable. Abdominal examination revealed a soft, non-tender abdomen; however, the uterus was palpable at approximately 12 weeks’ gestational size. Speculum examination demonstrated a long, closed cervix with a small amount of dark red blood in the vaginal vault and no active bleeding.

Serum β-hCG on presentation was elevated at 36,477 IU/L. Pelvic ultrasound demonstrated an anteverted uterus with a volume of 474 cc, an endometrial thickness of 11 mm, and no evidence of intrauterine or extrauterine pregnancy. Given the suspicion of GTN, β-hCG tumor marker was added. The level of 177 IU/L supported the differential of GTN.

**Figure 1:**
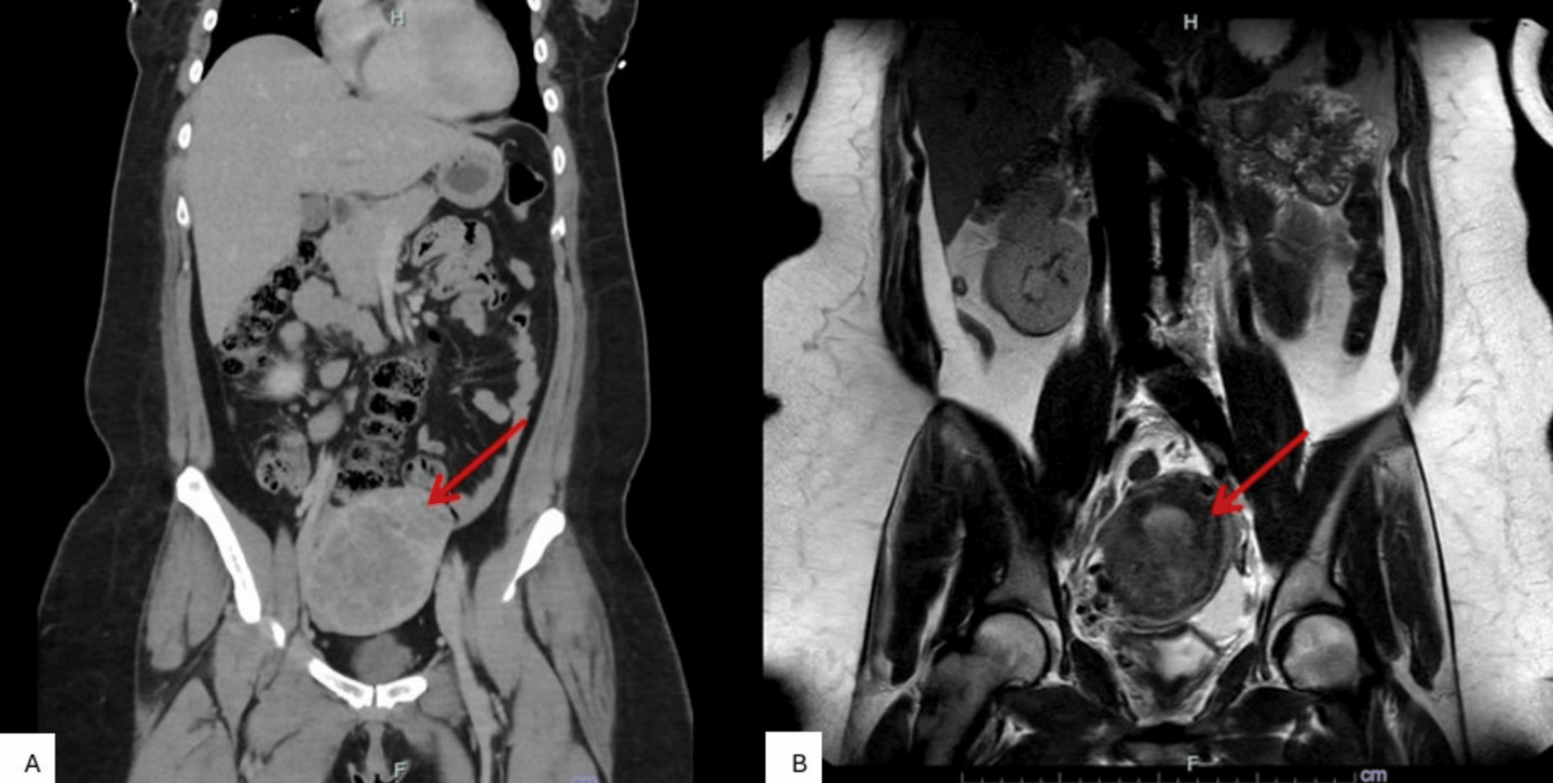
Cross-sectional imaging demonstrating a pelvic organ tumor. A. Coronal contrast-enhanced computed tomography of the abdomen and pelvis demonstrating a large, well-circumscribed heterogeneous mass within the pelvis (red arrow), extending into the lower abdomen. B. Coronal T2-weighted magnetic resonance imaging of the pelvis demonstrating the same lesion (red arrow) with heterogeneous signal intensity, providing improved delineation of the pelvic origin.

The patient underwent comprehensive staging, including contrast-enhanced computed tomography (CT) and magnetic resonance imaging (MRI). Multiple pulmonary nodules were present on chest CT, with the largest measuring 35 × 24 × 33 mm in the right upper lobe, located subpleurally. Additional nodules were noted adjacent to the oblique fissure and anterior lingula, each measuring 12 mm. Her pelvic CT revealed a heterogeneous fundal uterine mass measuring 82 × 66 × 92 mm (Figure 1A). Her pelvic MRI confirmed a large heterogeneous mass measuring 93 × 80 × 122 mm centered within the fundal myometrium (Figure 1B). Brain MRI showed no evidence of intracranial metastases. The patient was urgently referred to a tertiary gestational trophoblastic disease center for multidisciplinary team review.

Based on imaging and clinical findings, the patient was classified as FIGO Stage III (Table 1) with a WHO prognostic score of 9 (Table 2), consistent with high-risk GTN [1,7].

**Table 1:** FIGO staging system for gestational trophoblastic neoplasm. FIGO: International Federation of Gynecology and Obstetrics Reference: RCOG Green-top Guideline [7]

Stage	Description
I	Gestational trophoblastic tumor strictly confined to the uterine corpus
II	Gestational trophoblastic tumor extending to the adnexa or to the vagina but limited to the genital structures
III	Gestational trophoblastic tumor extending to the lungs, with or without genital tract involvement
IV	All other metastatic sites

**Table 2:** Modified WHO prognostic scoring system. β-hCG: β-human chorionic gonadotropin; WHO: World Health Organization Reference: Shahzadi et al. [1].

Risk factor	0	1	2	4
Age (years)	<40	>40	–	–
Antecedent pregnancy	Mole	Abortion	Term	–
Interval from last pregnancy (months)	<4	4 to 6	7 to 12	>12
Pre-treatment serum β-hCG (IU/L)	<1,000	1,000 to 10,000	10,000 to 100,000	>100,000
Largest tumor size (cm)	<3	3 to 4	>5	–
Site of metastasis	Lung	Spleen, kidney	Gastrointestinal tract	Brain, liver
Number of metastases	0	1 to 4	5 to 8	>8
Prior failed chemotherapy	None	–	1 drug	≥2 drugs

Comprehensive diagnostic investigations were performed. The patient underwent a hysteroscopic dilatation and curettage on September 24, 2025. However, the histopathology result showed secretory phase endometrium. Despite this, β-hCG remained elevated at 37,000 IU/L on October 1, 2025.

**Figure 2:**
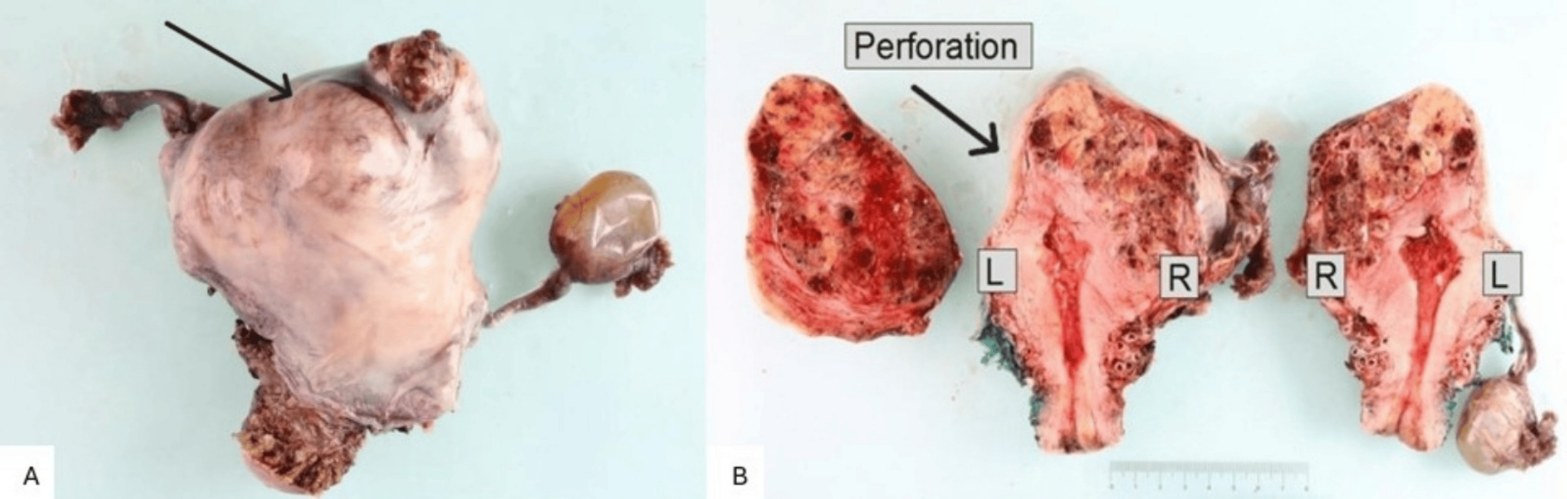
Gross pathological features of epithelioid trophoblastic tumor with uterine perforation. A. Intact uterus with adnexal structures showing a bulging mass arising from the uterine wall (arrow). B. Serially sectioned uterus demonstrating a hemorrhagic and necrotic tumor involving the uterine wall with focal perforation (arrow). L: left; R: right

**Figure 3:**
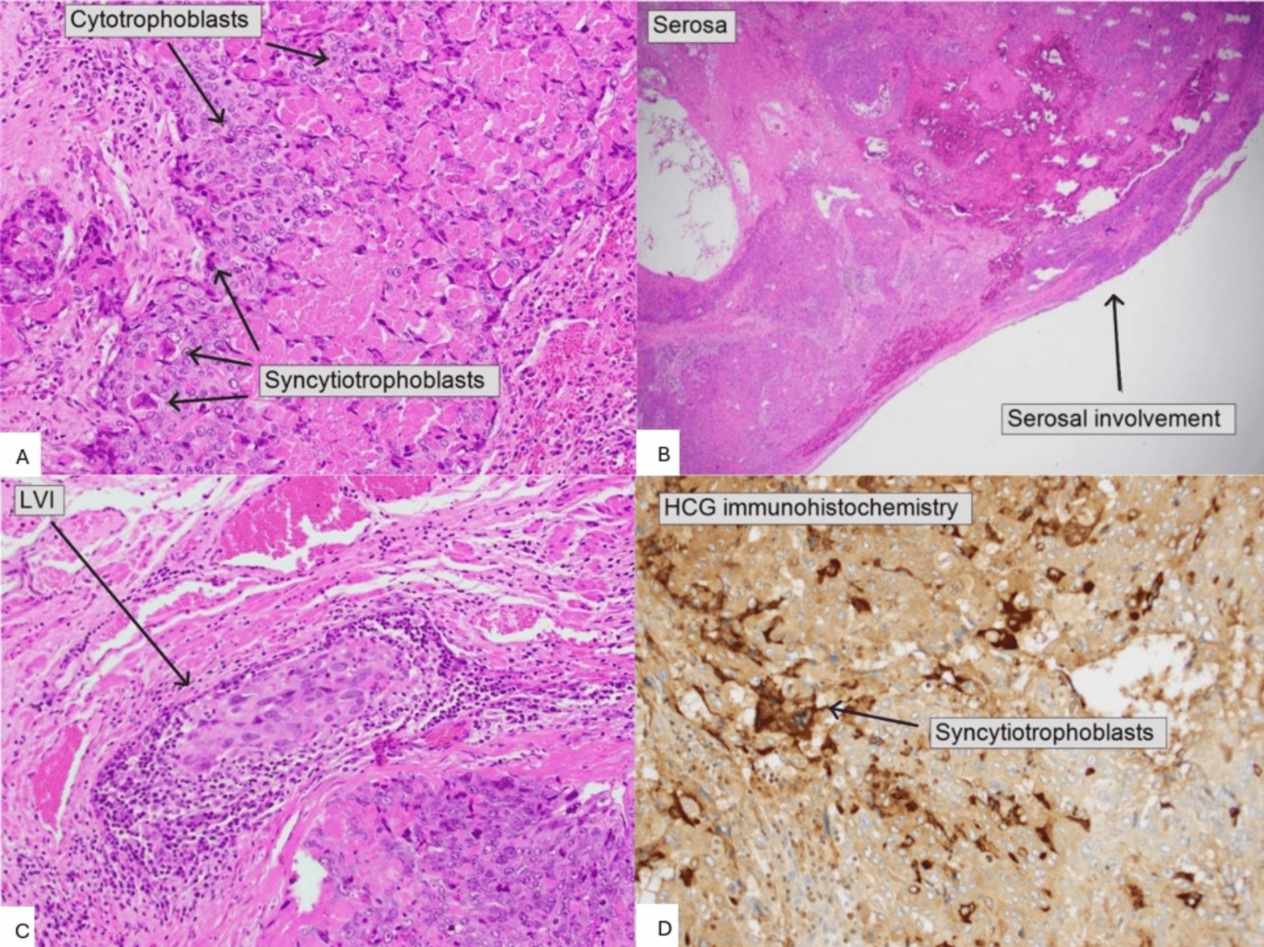
Histopathological and immunohistochemical features of epithelioid trophoblastic tumor. A: Hematoxylin and eosin stain showing nests of trophoblastic cells composed of cytotrophoblasts and syncytiotrophoblasts. B: Low-power view demonstrating tumor infiltration with serosal involvement. C: Lymphovascular invasion by tumor cells D: Immunohistochemistry for human chorionic gonadotropin showing cytoplasmic positivity in syncytiotrophoblastic cells. LVI: lymphovascular invasion; hCG: human chorionic gonadotropin

The patient subsequently underwent total laparoscopic hysterectomy and bilateral salpingectomy on  October 13, 2025, to obtain a definitive tissue diagnosis. The uterus measured 137 × 90 × 77 mm, weighed 390 g, and contained a 90 mm tumor originating in the right cornu, involving the fundus, and perforating the left side (Figure 2). Histologically, the mass consisted of scattered multinucleate syncytiotrophoblast-like cells, which seemed to be randomly distributed (Figure 3A). Lymphovascular space invasion was present (Figure 3B), and the tumor focally involved the uterine serosal surface (Figure 3C). Morphological features of choriocarcinoma were not evident. HCG immunohistochemistry was highly positive (Figure 3D). Immunohistochemistry demonstrated strong nuclear GATA3 and p63 positivity, diffuse cytoplasmic AE1/AE3, and a high proliferative index (~90%) on Ki-67, while SALL4, estrogen receptor, and progesterone receptor were negative (Table 3). These profiles supported a trophoblastic origin, excluded germ cell and epithelial tumors, and were consistent with a rare subtype of GTN, namely, ETT [11,12].

**Table 3:** Immunohistochemical profile of the tumor. Immunohistochemical analysis demonstrated diffuse positivity for GATA3, AE1/AE3, and p63. Ki-67 showed a high proliferative index of approximately 90%. The tumor cells were negative for SALL4, estrogen receptor (ER), and progesterone receptor (PR) [11,12].

Marker	Result	Staining pattern/Notes
GATA3	Positive	Strong nuclear staining
AE1/AE3	Positive	Diffuse cytoplasmic staining
p63	Positive	Nuclear staining
Ki-67	Positive (~90%)	High proliferative index
SALL4	Negative	No immunoreactivity
ER	Negative	No nuclear staining
PR	Negative	No nuclear staining

The patient was counselled regarding the diagnosis and initiated on multi-agent chemotherapy with EMA-EP (etoposide, methotrexate, actinomycin-D, etoposide, and cisplatin) on October 23, 2025 [4,9,10]. By day seven of chemotherapy, serum β-hCG declined to 8,192 IU/L. Her β-hCG levels during each clinical event are summarized in Table 4.

**Table 4:** Serial serum beta-hCG levels and key clinical events (2024–2025). β-hCG: β-human chorionic gonadotropin; IM: intramuscular; GP: general practitioner; ED: emergency department; EMA-EP: etoposide, methotrexate, actinomycin-D, etoposide, and cisplatin

Year	Date	Serum β-hCG (IU/L)	Clinical event
2024	March 12	623	Plateauing serum β-hCG between 400 and 600 over two weeks. Treated as ectopic pregnancy
	March 15	626	First IM methotrexate dose
	April 2	570	Second IM methotrexate dose
	April 12	599	Lost to follow-up
2025	April 15	13,700	GP visit for chronic vaginal bleeding
	August 18	36,477	ED presentation
	September 24	55,224	Rising level before surgery
	October 1	37,000	Pre-hysterectomy level
	October 15	9,604	Day three following hysterectomy
	October 23	9,027	Commencement of EMA-EP
	October 30	8,192	Day seven following chemotherapy

The patient tolerated the treatment well, with only mild fatigue and nausea managed conservatively. At present, the patient continues to receive chemotherapy under close monitoring with the aim of achieving complete remission. Multidisciplinary follow-up involving gynae-oncology, medical oncology, and radiology is ongoing to monitor treatment response and detect any potential recurrence.

## Discussion

GTN is a rare malignancy accounting for fewer than 1% of female reproductive tract tumors [1,2]. While most GTN subtypes respond well to chemotherapy with high cure rates even in metastatic disease, ETT presents unique challenges due to its rarity, relative chemoresistance, and potential for delayed diagnosis [3-5].

Clinically, GTN commonly presents with abnormal uterine bleeding and elevated serum β-hCG levels, which serve as essential diagnostic and surveillance biomarkers [3]. Although ETT typically produces lower β-hCG levels than choriocarcinoma, our patient exhibited a markedly elevated β-hCG of 36,477 IU/L, which is atypical for ETT [3-5]. This finding emphasizes that significantly elevated β-hCG does not exclude ETT and highlights the importance of maintaining diagnostic vigilance in atypical presentations. Unlike PSTT, where β-hCG elevation is usually mild, ETT may demonstrate variable β-hCG production, complicating early diagnosis [3-5].

GTN metastasizes most commonly to the lungs, vagina, and pelvis, with pulmonary involvement in approximately 80% of metastatic cases [6,7]. Metastases to less common sites, such as the liver and brain, carry worse prognoses and necessitate intensified management [8]. The detection of multiple pulmonary nodules in our case aligned with typical metastatic patterns.

Risk stratification using the WHO prognostic scoring system remains fundamental in guiding management [1]. A score ≥7 defines high-risk disease and necessitates multi-agent chemotherapy [1,7]. Our patient’s WHO score of 9 and FIGO Stage III disease (Tables 1, 2) indicated high-risk metastatic GTN and supported a multimodal treatment approach.

Definitive diagnosis relies on histopathological evaluation. ETT arises from chorionic-type intermediate trophoblasts and is characterized by nests and sheets of epithelioid cells with distinct cell borders, eosinophilic cytoplasm, geographic necrosis, and variable mitotic activity [3-5]. In ETT, immunohistochemistry typically shows strong nuclear p63 and GATA3 positivity, diffuse cytokeratin (AE1/AE3) expression, a high proliferative index (Ki‑67), and is negative for SALL4, ER, and PR [11,12]. In this case, the immuno-profile closely mirrored these features, with strong p63 and GATA3 staining, diffuse AE1/AE3 positivity, ~90% Ki‑67, and negative SALL4, ER, and PR, thereby supporting the diagnosis of ETT [11,12]. Together, these morphologic and immunophenotypic findings confirmed the diagnosis of ETT and were critical in distinguishing it from choriocarcinoma and PSTT, which differ in treatment responsiveness and prognosis [11,12].

Management strategies differ significantly between GTN subtypes. Low-risk GTN (WHO score 0-6) is typically treated with single-agent chemotherapy, most commonly methotrexate or actinomycin-D, achieving cure rates exceeding 90% [1,7]. High-risk GTN (WHO score ≥7) requires combination chemotherapy, with EMA-CO (etoposide, methotrexate, actinomycin-D, cyclophosphamide, and vincristine) serving as the standard first-line regimen. Alternative regimens such as EMA-EP are employed in cases of resistance or high tumor burden [4,7,9,10].

Unlike choriocarcinoma, which is highly chemosensitive, ETT demonstrates relative resistance to chemotherapy. Consequently, surgical resection, most commonly hysterectomy, plays a central role, particularly in localized disease [4-10]. Surgery provides both definitive diagnosis and local tumor control. In metastatic or high-risk cases, adjuvant multi-agent chemotherapy is recommended to address systemic disease.

In our patient, a hysterectomy was performed for diagnostic clarification in the setting of persistently elevated β-hCG and imaging findings suspicious for neoplasm. Given the high-risk score and pulmonary metastases, adjuvant multi-agent chemotherapy was initiated postoperatively. The patient demonstrated a favorable biochemical response, underscoring the importance of early surgical intervention combined with systemic therapy in selected cases of metastatic ETT.

Overall, ETT carries a less favorable prognosis compared with other GTN subtypes due to delayed diagnosis and reduced chemosensitivity. Early recognition, accurate histopathological and immunohistochemical characterization, appropriate risk stratification, and timely multimodal management are essential to optimize outcomes. Close surveillance with serial β-hCG monitoring remains critical for early detection of recurrence and assessment of treatment response [11,12].

## Conclusions

GTN, although rare, is highly treatable when diagnosed promptly. ETT presents distinct diagnostic and therapeutic challenges due to its relative chemoresistance and potential for metastatic spread. The WHO prognostic scoring system provides essential risk stratification, guiding the use of aggressive multimodal therapy. Surgical intervention remains central for both diagnostic confirmation and local disease control in ETT. Multidisciplinary management, combining surgery with multi-agent chemotherapy, can achieve remission, as demonstrated in this case. Ongoing surveillance with serial serum β-hCG monitoring is critical for early detection of recurrence and long-term disease control.
